# Cohort Profile Update: The Harmonised Cognitive Assessment Protocol Sub-study of the English Longitudinal Study of Ageing (ELSA-HCAP)

**DOI:** 10.1093/ije/dyaa227

**Published:** 2020-12-28

**Authors:** Dorina Cadar, Jessica Abell, Fiona E Matthews, Carol Brayne, G David Batty, David J Llewellyn, Andrew Steptoe

**Affiliations:** 1 Department of Behavioural Science and Health, University College London, London, UK; 2 Population Health Sciences Institute, Newcastle University, Newcastle, UK; 3 Department of Public Health and Primary Care, University of Cambridge, Cambridge, UK; 4 Department of Epidemiology and Public Health, University College London, London, UK; 5 School of Biological & Population Health Sciences, Oregon State University, Corvallis, OR, USA; 6 Medical School, University of Exeter, Exeter, UK; 7 Alan Turing Institute, London, UK

## The original cohort

The English Longitudinal Study of Ageing (ELSA) is an ongoing nationally representative sample of individuals aged 50 and older living in England.^1^ The study started in 2002, and participants are reassessed every 2 years with face-to-face interviews and self-completion questionnaires. Biomedical assessments take place every 4 years with a home visit from a study nurse who assesses functional capacity and anthropometry and takes blood samples for the extraction of biomarkers and DNA. More than 18 000 people have taken part in the study since its inception, and the data currently available span over 16 years (wave 9 took place in 2018/19). Refreshment samples of new participants have been recruited at waves 3, 4, 6, 7 and 9 to maintain the age profile and ensure that the study remains representative of the English population aged 50 and over. ELSA tracks multiple and complex characteristics of the same individuals as they move through middle age to older age and has collected a wide range of clinical, biological, psychological, economic and social measures, including biomarker and genetic data across the main waves. The study is a collaboration between epidemiology and behavioural science (University College London), economics (Institute for Fiscal Studies), social science (University of Manchester), clinical medicine (Norwich Medical School) and survey specialists (NatCen Social Research). For up-to-date information, see the study website [https://www.elsa-project.ac.uk/]. ELSA data are archived with the UK Data Service [https://ukdataservice.ac.uk] within a few months of completion of each wave, and are accessed by researchers and policy analysts around the world. The study informs policy across many aspects of ageing, including health and social care, retirement and pensions policy and social and civic participation. Internationally, ELSA is one of the sister studies of the Health and Retirement Study (HRS) in the USA, and part of a growing network of longitudinal population studies of ageing around the world. These surveys not only provide data for individual countries but also offer the opportunity for cross-national comparisons of harmonized datasets. For more information, see the Gateway to Global Ageing Data website [https://g2aging.org/].

## Ethics Approval

The ELSA-HCAP Sub-study received ethical approval from the South Central-Berkshire National Health Services (NHS) Research Ethics Committee and was conducted in accordance with the ethical standards of the Declaration of Helsinki. Informed verbal consent was obtained from all participants or their guardians. For more information see Supplementary material available as Supplementary data at *IJE* online.

## What is the reason for the new data collection?

One of the key epidemiological challenges in the 21st century is that non-communicable diseases, such as cardiovascular disease, stroke and dementia, have become the leading causes of mortality and morbidity.^2^ Among these, dementia represents one of the most debilitating conditions, with an increased prevalence in individuals aged 65 years and older and double risk with every 5 years’ increase in age after that. Attributable pathologies accounting for dementia at death suggest that age, brain atrophy, total volume loss, vascular changes and disease-related proteins such as TDP-43 and alpha-synuclein all contribute, with Alzheimer’s type pathology alone accounting for only around 20% of cases.^3^ Early detection and prevention are crucial but challenging, considering the interplay of the established risk factors operating at various points across the life course.^4^

The operationalization of diagnostic criteria for the ever-changing concepts of mild cognitive impairment and dementia, as well as their diagnostic boundaries, are complex. Yet in most cases, diagnosis involves a medical history and reported or measured changes in cognitive function, as well as behavioural and functional impairments that allow clinicians to gauge the severity of the symptoms presented. A significant challenge is to define what constitutes the normal spectrum of cognitive ageing in contrast to cognitive impairment while taking into consideration specific population norms.^5^ Despite our ability to identify certain brain pathologies *in vivo* using neuroimaging and biomarkers,^6^ a precise diagnostic algorithm remains problematic. The most significant difficulties encountered in diagnosing dementias stem principally from their shared heterogeneous classifications, influenced by multiple and mixed pathologies. Modern conceptualizations highlight preclinical stages reflecting changes that have already taken place in the brain before marked symptoms emerge.^7^ Crucially, individuals who are clinically diagnosed are only a subset of those with cognitive impairment and dementia, and it is estimated that dementia remains undetected in almost 50% of primary care patients in the UK,^8^ with even higher rates in other countries. In the absence of a single measurement instrument for dementia identification and classification, international efforts are being made to implement standardized neurocognitive assessments in various population studies.

The implementation of the international Harmonised Cognitive Assessment Protocol (HCAP) in the family of studies associated with the Health and Retirement Study offers an opportunity for investigating, in a comparable manner, measures relevant to dementia diagnosis including cognitive, sensory and psychological performance as well as functional abilities in large representative population samples of older adults in both high- and middle-income countries. The overall aim of HCAP is to ascertain and investigate mild cognitive impairment and dementia across the general population worldwide. HCAP involved much more detailed assessments of cognitive impairment and its correlates than was possible in the main waves of ELSA and so was carried out as a sub-study. ELSA-HCAP provides opportunities for identifying potential predictors of cognitive impairment and dementia, as well as the consequences of dementia, in the context of the longitudinal framework of ELSA main waves.

## What will be the new areas of research?

The ELSA-HCAP protocol involves an extensive range of cognitive measures coupled with informant interviews on a stratified sub-sample of ELSA participants aged 65 and older. These cognitive measures will be included in an international algorithm aimed to classify dementia, mild cognitive impairment and healthy cognitive function in population studies, with benchmarking against the Cognitive Function and Ageing Study (CFAS), the Aging Demographics and Memory Study (ADAMS) and other studies of cognitive ageing and dementia around the world.

The HCAP instrument aims to use international algorithmic approaches to evaluate the prevalence of neurocognitive disorders in people aged over 65 years within each participating country including England, USA, Mexico, South Africa, China and India. Currently, the HCAP Network research group is developing a diagnostic algorithm that will make use of HCAP respondent and informant data to assign a research diagnosis of normal, mild cognitive impairment or dementia.

The HCAP project is being carried out in several countries around the world, and as a result, the international implementation of these assessments offers the opportunity for cross-national investigations and comparisons of the biological, medical, social and environmental factors that affect the risk of neurocognitive impairments. Therefore, HCAP represents a promising international platform for investigating and tracking changes in the prevalence of mild cognitive impairment and dementia over time, facilitating country-specific investigations of medical care systems and social policy interventions.

## Who is in the cohort?

Participants were selected for the ELSA-HCAP Sub-study if they were an ELSA core member aged 65 and over at the start of fieldwork in January 2018 (born before 1 January 1953) and had completed an ELSA interview in person at either wave 8 (2016–17) or wave 7 (2014–15). Core members living in care or nursing homes were also eligible, if they had the capacity to consent, or if a consultee/family member agreed to participate on their behalf. Further selections were made based on a sample procedure related to their previous cognitive performance using the modified Telephone Interview Cognitive Screening (mTICS)^9^ and a diagnosis of Alzheimer’s disease or dementia previously reported (waves 1–8). Three groups were defined using the thresholds on the mTICS 27 items scale: group 1—low cognition (≤6 mTICS27 score) and/or a diagnosis of Alzheimer’s disease or dementia; group 2—moderate cognition (7–11 mTICS27 score) and had never reported a diagnosis of Alzheimer’s disease or dementia; group 3—normal cognition (≥12 mTICS27 score) or unknown for those with missing data on mTICS scores at most recent ELSA waves. ELSA-HCAP was administered to participants across all the range of cognitive abilities, but we oversampled those identified as having low cognitive scores in the most recent ELSA waves. Core members who had proxy interviews (indicating an inability to participate themselves) at both wave 7 and wave 8 were not eligible for ELSA-HCAP.

### ELSA-HCAP dress rehearsal and fieldwork

The fieldwork for ELSA-HCAP took place between January 2018 and April 2018, between the ELSA main waves 8 and 9 (Supplementary Figure S1, available as Supplementary data at *IJE* online). To test the design, materials and procedure for the ELSA-HCAP Sub-study, a dress rehearsal was conducted between August and September 2017. For more information, see Supplementary material, available as Supplementary data at *IJE* online.

### ELSA-HCAP participants selection and response rates

**Figure 1:**
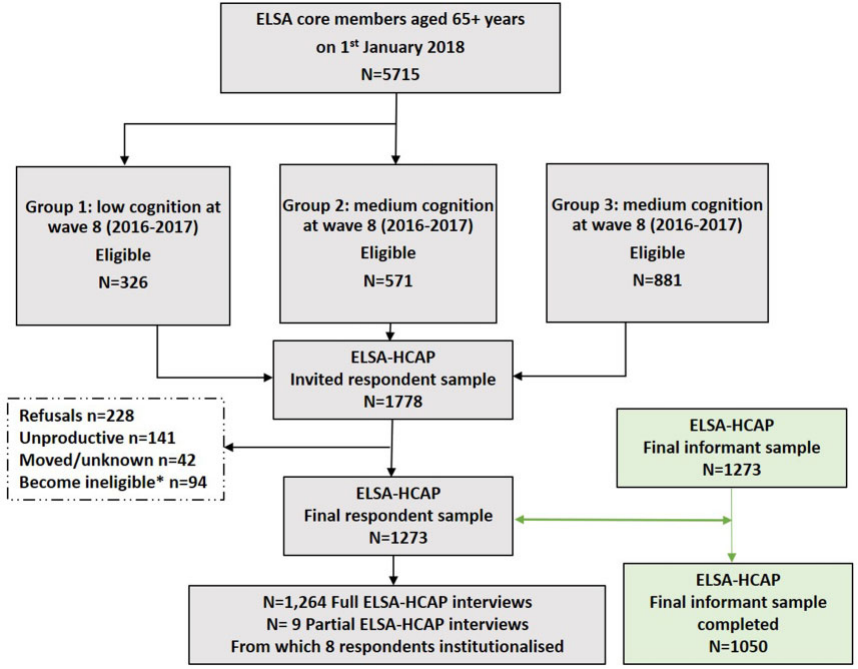
The flow of respondents and informant selection through the study in the English Longitudinal Study of Ageing Harmonised Cognitive Assessment Protocol (ELSA-HCAP)

Based on the eligibility criteria, around 1800 individuals were sampled for ELSA-HCAP, with the expectation of a 60% response rate. Around half (*n* = 900) were sampled from cognitive groups 1 (low) and 2 (moderate). Since the number of people eligible for group 1 (*n* = 334) was small, all eligible members were invited, whereas for those eligible in group 3 (*n* = 4 610) only 1 in 5 people were invited. The response rate for the ELSA-HCAP study was higher than anticipated. From the 1684 eligible cases, 1273 completed the face-to-face interview, representing a final response rate of 75.6% (Figure 1). Table 1 shows that response rates were generally similar across age and sex, but with a slightly higher response within the 65–69 age group and lower in the 85+ age group.

**Table 1:** The response among ELSA-HCAP respondents and informants (eligible and completed interviews)

		ELSA-HCAP respondent			ELSA-HCAP informant	
Group	Category	Eligible	Completed interviews		Completed interviews	
		*n*	*n*	%	*n*	%
Sex	Male	742	573	77.2	371	–
	Female	942	700	74.3	679	–
Age	<65	–	–	–	375	–
	65–69	376	301	80.1	174	–
	70–74	391	299	76.5	183	–
	75–79	339	259	76.4	154	–
	80–84	328	249	75.9	108	–
	85–89	162	114	70.4	27	–
	90 and over	88	51	57.9	–	–
	Missing	–	–	–	23	–
Cognitive grouping selection	1: Low	282	179	63.5	154	86.0^*^
	2: Moderate	540	419	77.6	364	86.9^*^
	3: Normal	862	675	78.3	618	91.5^*^
		1684	**1273**	75.6	**1050**	82.5^*^

ELSA-HCAP: English Longitudinal Study of Ageing-Harmonised Cognitive Assessment Protocol.

* Percentage response rates from the completed number of respondents.

1273 represents the total sample of ELSA-HCAP respondents and 1050 represents the total sample of ELSA-HCAP informants.

### ELSA-HCAP informants selection and response rates

A total of 1050 informant interviews were conducted, representing a response rate of 82.5% of all the eligible sample (*n *= 1273) contacted. Of these, 194 (18.5%) informant interviews were completed by telephone, and 856 (81.5%) by self-completion questionnaire. The informant relationships with the respondent included spouse/partner (56%), child (22%), grandchild (1%), sibling (3%), parent (3%), friend (10%), carer (0.3%), neighbour (1%) or other (3%). A small number refused to answer (0.4%). The average period an informant reported knowing the respondent was 43 years [standard deviation (SD) = 18; range = 1–82]. Table 2 also shows the response rates among the informant sampling groups (eligible and completed interviews), which were similar across age and sex, with slightly lower rates for informants aged 80 and over.

**Table 2:** The battery of tests included in the ELSA-HCAP respondent and informant interviews with the correspondence between ELSA main waves

	Neuropsychological domain	ELSA-HCAP	ELSA main waves
**ELSA-HCAP respondent interview**			
Mini-Mental State Examination (MMSE)	Multiple	✓	X
HRS Telephone Interview for Cognitive Status (HRS-TICS)		✓	✓
CERAD word list recall-immediate	Memory	✓	✓ (alternative list)
Retrieval fluency	Language	✓	✓
Letter cancellation	Visuospatial	✓	✓
Backward count	Attention	✓	✓
10/66 (Community Screening Instrument for Dementia, CSI-D)	Executive functioning	✓	X
CERAD word list recall–delayed	Memory	✓	✓ (alternative list)
East Boston memory test–immediate	Memory	✓	x
Wechsler memory scale-IV^a^–immediate	Memory	✓	X
CERAD word list recognition	Memory	✓	X
Constructional praxis–immediate	Memory	✓	X
Symbol-digit modalities test	Executive functioning	✓	X
Constructional praxis–delayed	Visuospatial	✓	X
Wechsler memory scale-IV–delayed	Memory	✓	X
East Boston memory test–delayed	Memory	✓	x
Wechsler memory scale-IV–recognition	Memory	✓	X
Number series	Executive functioning	✓	X
Raven’s standard progressive matrices	Executive functioning	✓	X
Trail making A & B	Executive functioning	✓	X
Center for Epidemiological Studies Depression Scale (CES-D)	Depression	✓ (11 items)	✓ (8 items)
Smell test	Olfaction	✓	X
**ELSA-HCAP informant interview**	**Functional domain**		
Jorm informant questionnaire on cognitive decline in the elderly (IQCODE)	Informant evaluation	✓	✓
Blessed dementia rating scale-part 2	Informant evaluation	✓	x
HRS activities questionnaire	Informant evaluation	✓	x
Community Screening Instrument for Dementia (CSI-D) cognitive activities questionnaire	Informant evaluation	✓	x
10/66 dementia research group informant questionnaire	Informant evaluation	✓	X
Blessed dementia rating scale–part 1	Informant evaluation	✓	X

ELSA-HCAP: English Longitudinal Study of Ageing-Harmonised Cognitive Assessment Protocol; HRS, Health Retirement Study; CERAD, Consortium to Establish a Registry for Alzheimer’s Disease.

a Wechsler memory scale-IV story administered in this study was ‘Anna Thompson’.

### Sample weights

A weighting procedure was derived for the ELSA-HCAP Sub-study in order to adjust for non-response bias within each of the three cognition groups, especially for the low cognition group, which had the lowest response rate. It involved three components: design weights, non-response weights and a calibration procedure accounting for differential selection probabilities and adjusting for non-response. The final weight for this sub-study represents a combination of the design and non-response weights, which is made available to the users with the ELSA-HCAP data (Supplementary data and Supplementary Table S1, available at *IJE* online).

## How often have they been followed up?

At the time of this report, only wave 1 of the ELSA-HCAP Sub-study has been completed. However, participants will continue to be included in the ELSA main waves, so it is expected that they will be followed up as part of the study. It is planned that ELSA will continue with assessments every 2 years, nurse visits for biomarkers measurements every 4 years and linkage with registry data, including mortality statistics.

### Data quality

To ensure data quality, two researchers scrutinized the data collected, by comparing the results between various tests and the overall performance on the Mini-Mental State Examination (MMSE). The concordance between the distributions of ELSA-HCAP cognitive scores with those published in similar age-matched population studies was also examined. When outliers were identified (±3 standard deviations from the mean), further checks of the paper tests and interview records were made, correcting any errors in data entry (*n* = 48).

## What has been measured?

The HCAP comprises two interviews, one with the respondent and one with an informant nominated by the respondent.

*The HCAP respondent interview* covers a broad range of cognitive domains (memory, language, executive function, psychomotor speed, problem solving and numeracy) known to be affected by the ageing process. Table 2 summarizes these tests, the majority of which are new to ELSA, although they all are well-established neurocognitive assessments such as Mini-Mental State Examination (MMSE), CERAD (Consortium to Establish a Registry for Alzheimer’s Disease), Word List Memory, Backward Counting Task, Community Screening Interview for Dementia, Logical memory (East Boston Memory Test and ‘Anna Thompson’ story of the Wechsler Memory Scale), CERAD Constructional Praxis (shape drawing), Symbol Digit Modalities Test, HRS Number Series, Raven’s Standard Progressive Matrices Test, and Trail Making. For more detail and references, see Supplementary Table S2, available as Supplementary data at *IJE* online. The 11-item version of the Center for Epidemiologic Studies Depression Scale (CES-D) was also administered, together with an optional test of olfaction (smell), evaluating odour identification and detection. The duration of the ELSA-HCAP respondent interview was approximately 1 h (range 17.6–186.2 min, median 72.8 min).

*The ELSA-HCAP informant interview* was conducted with a nominated family member or knowledgeable friend who was asked to describe the general health of the respondent and evaluate any changes in their cognitive abilities. The interview measured the functional status of the respondent, such as their ability to engage in activities of daily living and social, and leisure activities. It also determined whether the respondent had been previously diagnosed with a stroke, Alzheimer's disease, other forms of dementia or Parkinson’s disease. Further measures included were the Informant Questionnaire on Cognitive Decline in the Elderly, Blessed Dementia Rating Scale, Community Screening Instrument for Dementia (CSI-D) Cognitive Activities Questionnaire, 10/66 Dementia Research Group Informant Questionnaire and HRS Activities Questionnaire, comprising questions related to the amount of time spent watching TV, reading, doing household chores, yoga or other exercises (see Table 2 and Table S2). The length of time for which the informant had known the respondent was also recorded. The informant interview was completed either via a paper self-completion questionnaire or by a telephone interview using computer-assisted telephone interviewing. The ELSA-HCAP informant interview took approximately 20–25 min to complete.

## What has it found? Key findings and publications

The ELSA-HCAP Sub-study provides a detailed neuropsychological and clinical assessment of a selected sample of individuals aged 65 and over which can be extrapolated to the rest of the ELSA population and, by extension, to those living in the community in England. Table 3 shows the sociodemographic characteristics of the ELSA-HCAP respondent sample. Gender differences were observed in educational attainment and wealth, with more men attaining a higher level of education and achieving higher wealth than women. Men were more likely than women to be married or living with a partner and to smoke or drink frequently. In contrast, women were more likely to feel lonely and experience difficulties in the Instrumental Activities of Daily Living, particularly using a map, shopping or working around the house/gardening. Table 4 presents the weighted means and standard deviation for each cognitive test included in the ELSA-HCAP respondent interview. The performance of men was on average better than women on the MMSE, verbal fluency, backwards counting, constructional praxis, logical reasoning (number series) and solving intelligence tests (Ravens). Women outperformed men in memory and visual-spatial scanning abilities (letter cancellation).

**Table 3.** Sociodemographic, lifestyle and health characteristics of the ELSA-HCAP sample

	Overall sample	Men	Women	
	Mean (SD)/%	Mean (SD)/%	Mean (SD)/%	*P*-value
*n*/variables	1273	573 (45%)	700 (55%)	
Age	75.3 (6.6)	75.1 (6.4)	75.4 (6.7)	0.83
Educational attainment				≤0.001
No qualification	37.5%	33.2%	40.1%	
Education to age 16	17.2%	15.7%	18.5%	
Education to age 18	33.5%	34.4%	32.8%	
Degree	11.8%	16.8%	7.8%	
Wealth				≤0.001
Lowest	33.6%	28.2%	38.1%	
Medium	33.1%	33.4%	32.9%	
Highest	33.3%	38.5%	29.3%	
Geographical region				0.91
North	5.8%	5.8%	5.9%	
Yorkshire	10.5%	10.3%	10.6%	
East Midlands	13.0%	12.6%	13.4%	
East Anglia	11.1%	10.8%	11.3%	
South East	11.9%	12.0%	11.7%	
South West	12.6	13.8%	11.6%	
West Midlands	7.1%	7.0%	7.1%	
North West	17.1%	18.0%	16.4%	
Wales	11.0%	9.8%	12%	
Depressive symptoms^a^	2.9%	2.3%	3.4%	0.22
Poor eyesight	4.1%	4.1%	4.2%	0.91
Poor hearing	7.0%	8.2%	6.0%	0.13
Poor olfaction	7.3%	7.5%	7.2%	0.83
Poor general health	10.1%	10.0%	10.1%	0.86
Cardiovascular condition	4.8%	5.8%	4.0%	0.15
Diabetes	5.3%	5.4%	5.2%	0.84
Hypertension	15.0%	15.0%	15.0%	0.99
Stroke	2.1%	2.8%	1.6%	0.13
Cancer	4.8%	5.2%	4.4%	0.51
Respiratory disease	4.6%	5.8%	3.6%	0.06
Activities of daily living				
Difficulty dressing	11.8%	11.3%	12.2%	0.65
Difficulty walking	6.9%	5.9%	7.8%	0.24
Difficulty bathing	10.9%	9.3%	12.2%	0.10
Difficulty eating	2.0%	2.6%	1.4%	0.13
Difficulty getting out of bed	4.9%	4.4%	5.3%	0.44
Difficulty using toilet	3.9%	3.3%	4.4%	0.30
Instrumental activities of daily living				
Difficulty using map	7.2%	5.6%	8.5%	0.05
Difficulty recognizing danger	1.7%	2.1%	1.4%	0.37
Difficulty preparing a meal	6.7%	5.9%	7.3%	0.33
Difficulty shopping	12.9%	9.6%	15.6%	≤0.001
Difficulty making calls	4.2%	4.9%	3.6%	0.25
Difficulty communicating	4.7%	5.4%	4.2%	0.29
Difficulty taking medication	4.5%	4.2%	4.7%	0.64
Difficulty working around	17.8%	12.9%	21.8%	≤0.001
house/gardening				
Difficulty managing money	7.2%	6.6%	&0 .6%	0.51
Sedentary lifestyle	10.6%	10.3%	11.0%	0.67
Smoking	8.2%	8.6%	7.9	≤0.001
Drinking (daily)	20.7%	27.2%	15.2%	≤0.001
Married/living with partner	64.6%	75.0%	54.3%	≤0.001
Feels lonely	6.8%	4.3%	9.1%	≤0.001

ELSA-HCAP: English Longitudinal Study of Ageing-Harmonised Cognitive Assessment Protocol.

a Depressive symptoms were ascertained using the threshold of 9 on the 11-item version of the Centre for Epidemiologic Studies Depression Scale (CES-D);

**Table 4:** Descriptive statistics presenting weighted means and standard deviation of the cognitive tests included in the ELSA-HCAP respondent interview

	Overall sample		Men		Women		
Cognitive test	*n*	Mean (SD)	*n*	Mean (SD)	*n*	Mean (SD)	*P*-value
MMSE	1273	26.8 (3.3)	573	27.2 (3.0)	700	26.5 (3.5)	≤0.001
HRS-TICS	1271	2.8 (0.5)	572	2.8 (0.5)	699	2.7 (0.6)	0.10
CERAD immediate	1263	18.4 (5.1)	569	17.7 (4.9)	694	18.9 (5.2)	≤0.001
CERAD delayed	1264	5.5 (2.6)	568	5.2 (2.4)	696	5.8 (2.6)	≤0.001
CERAD recognition	1261	18.6 (2.5)	567	18.6 (2.2)	694	18.5 (2.7)	0.57
Retrieval fluency	1270	19.2 (8.5)	572	20.2 (8.8)	698	18.3 (8.2)	≤0.001
Letter cancellation	1186	269.1 (84.5)	542	258.6 (76.3)	644	278.3 (85.5)	≤0.001
Backwards counting	1253	31.5(10.9)	563	33.2(10.8)	690	30.1(10.9)	≤0.001
CSID	1271	4.0 (0.3)	572	4.0 (0.2)	699	3.9 (0.3)	0.01
EBMT story							
immediate	1264	4.1 (1.5)	568	4.1 (1.5)	696	4.2 (1.5)	0.43
delayed	1217	2.9 (1.9)	546	2.8 (1.9)	671	2.9 (1.9)	0.22
Wechsler story							
immediate	1254	9.3 (4.7)	565	9.2 (4.5)	689	9.4 (4.8)	0.28
delayed	1210	7.2 (4.7)	544	7.0 (4.6)	666	7.4 (4.8)	0.17
recognition	1222	11.3 (2.8)	549	11.4 (2.7)	673	11.3 (2.9)	0.57
CERAD Praxis							
drawing	1250	9.5 (1.8)	564	9.9 (1.6)	686	9.2 (1.9)	≤0.001
delayed recall	1161	7.8 (2.8)	531	8.3 (2.7)	630	7.3 (2.8)	≤0.001
Symbol digit	1196	33.8 (12.6)	546	33.7 (11.9)	650	33.9 (13.1)	0.64
Number series	1153	530.4 (31.4)	528	536.3 (29.9)	625	525.3 (31.7)	≤0.001
Ravens	1258	13.6 (3.5)	567	14.1 (3.2)	691	13.2 (3.7)	≤0.001
Trail making A	1209	55.0 (34.9)	548	53.9 (35.8)	661	55.9 (34.1)	0.33
Trail making B	1038	116.1 (57.5)	487	115.1 (54.1)	551	116.9 (60.5)	0.65

ELSA-HCAP: English Longitudinal Study of Ageing-Harmonised Cognitive Assessment Protocol. MMSE, Mini-Mental Status Examination; HRS-TICS, Health Retirement Study-Telephone Interview for Cognitive Status; CERAD, Consortium to Establish a Registry for Alzheimer’s Disease; CSI-D, Community Screening Instrument for Dementia; EBMT, East Boston Memory Test.

The longitudinal nature of ELSA provides opportunities for the investigation of precursors of cognitive impairment and dementia over the 16 years of data, including the role of demographic, economic, social, cognitive, behavioural, biological and health factors. These in-depth phenotype data provide important avenues to address the timing, duration and multifactorial nature of the many biological, medical and social factors that influence the onset of clinical symptoms of dementia.

There have been >30 peer-reviewed articles published using ELSA data on cognitive functioning and dementia outcomes, covering a wide range of issues: see [https://www.elsa-project.ac.uk/publications]. However, these analyses preceded the implementation of HCAP Sub-study and we have, therefore, outlined a selection of findings to illustrate the broad spectrum of factors that can be related to cognitive functioning and dementia.

In ELSA, information about dementia has to date been captured through a self-reported physician diagnosis of dementia or Alzheimer’s disease and informant (proxy interviews) evaluations via the Informant Questionnaire on Cognitive Decline in the Elderly.^10^ A proxy interview is usually carried out with a nominated person (e.g. a family member) when the core member is not able or declines to take part but consents that someone else can complete a shorter interview on their behalf. These measures have been used in a number of studies to model future trends of dementia incidence^11^ and to investigate socioeconomic determinants^12^ and other modifiable risk factors associated with dementia^13^ such as social support,^14^ loneliness,^15^ digital literacy^16^ and social and cultural engagement,^17^ as well as a range of non-modifiable risk factors such as stroke,^18^ sensory impairments,^19^ physical capability^20^ and frailty.^21^ Over the different waves of data collection, an array of cognitive tests has been administered including measures of verbal memory, orientation, verbal fluency and letter cancellation, in addition to the assessment of basic cognitive abilities such as numerical ability and literacy. These have been used to investigate the relationship between cognitive performance and depressive symptoms,^22^ level of neighbourhood deprivation,^23^ diabetes,^24^ sensory impairments^25^ and mortality.^26^ Furthermore, international comparisons of cognitive performance have been conducted across middle-aged and older adults in England and the USA.^27^

## What are the main strengths and weaknesses?

The ELSA-HCAP used identical measures to the HRS-HCAP, providing enhanced opportunities for international comparisons of the broader biopsychosocial context of cognitive impairment and algorithmic approaches to dementia definition. Furthermore, the ELSA-HCAP battery of tests has considerable overlap with other relevant studies such as the Cognitive Function and Ageing Studies (CFAS),^28^ Aging, Demographics, and Memory Study (ADAMS), Rush Memory and Aging Project^29^ and 10/66 dementia study.^30^

Several limitations should be acknowledged. The ELSA-HCAP Sub-study is currently cross-sectional, and a follow-up would be valuable to assess patterns of change in this detailed extended neuropsychological battery. Adding neuroimaging to the study would also greatly enhance the research possibilities of discriminating structural brain changes related to healthy ageing from the level of abnormal atrophy and neuronal loss associated with various neurocognitive disorders. Combined with detailed functional imaging, highlighting specific biochemical and molecular processes could inform and refine diagnostic accuracy. An important limitation is that the study sample is predominantly of White European ancestry. ELSA was designed to be representative of the population of older people in England in 2002 when only 3.2% of the older population in England were from ethnic minorities, according to the national census. Last, the number of ELSA-HCAP participants who were institutionalized at the time of the interview was very low (*n *= 8), restricting generalization to more fragile sectors of the population.

Some features that make the HCAP Sub-study distinctive arise from it being embedded in ELSA. Several biomarkers have been assessed in the study sample every 4 years since 2004. Study nurses have collected venous blood for the determination of lipids (total cholesterol, HDL cholesterol, triglycerides), glucose and glycated haemoglobin (HbA1c), inflammatory markers including C-reactive protein, fibrinogen, and white blood cell counts, ferritin and haemoglobin, insulin-like growth factor 1 (IGF1), vitamin D and other biomarkers that could be analysed longitudinally in relation to many ageing outcomes.

In addition to economic, psychosocial and health measures, genome-wide genotyping was carried out on 7412 ELSA participants of European ancestry, using the same Illumina HumanOmni2.5 Beadchip as used in the HRS. Polygenic risk scores for Alzheimer’s disease (AD) and dementia have been created using results from the genome-wide association study conducted by the International Genomics of Alzheimer’s Project, and have been made available through the UK Data Service. This offers a rich resource of data for various analyses involving genome-wide gene-environment or gene-gene interactions as well as the potential for Mendelian randomization analyses and sub-phenotyping patient-stratification investigations.

Moreover, because participants in ELSA-HCAP will be followed up in future waves of data collection, it will be possible to quantify the consequences of severe cognitive impairment for family income and expenditure, financial decision making, social connectivity, mental well-being and physical health. This information has the potential to strengthen the evidence base for health and social care provision to this vulnerable sector of the population, and other vital policy initiatives related to older fragile men and women.

## Can I get hold of the data? Where can I find out more?

The ELSA-HCAP data, including the individual items and the derived scores for each of the respondent and informant interviews, have been made available via UK Data Service [https://ukdataservice.ac.uk]. The ELSA-HCAP study number (SN) is 8502; http://doi.org/10.5255/UKDA-SN-8502-2. The main ELSA study is SN 5050 and is held under [http://doi.org/10.5255/UKDA-SN-5050-17]. The person to contact for the ELSA-HCAP study is Dr Dorina Cadar (e-mail: d.cadar@ucl.ac.uk).

**Profile in a nutshell**

- The Harmonised Cognitive Assessment Protocol Sub-study of the English Longitudinal Study of Ageing (ELSA-HCAP), comprising 1273 men and women aged 65 and older, was set up in 2018 to ascertain the prevalence of neurocognitive disorders such as cognitive impairment and dementia and to investigate the risk factors and regional variations in these conditions across England.
- The ELSA-HCAP protocol represents a more extensive range of cognitive measures than those collected in regular waves of the English Longitudinal Study of Ageing (ELSA), covering a broad range of cognitive domains such as memory, language, executive function, psychomotor speed, problem solving and numeracy, which are known to be affected by the ageing process.
- The performance of men was on average better than women on the Mini-Mental State Examination, verbal fluency, backwards counting, constructional praxis, logical reasoning (number series), and solving intelligence tests (Ravens). Women outperformed men in memory and visual-spatial scanning abilities (letter cancellation). However, it should be noted that levels of educational attainment were higher among men than women.
- The harmonized cognitive measures will contribute to an international algorithm aimed at classifying mild cognitive impairment and dementia, benchmarking against the Cognitive Function and Ageing Study (CFAS), Aging Demographics and Memory Study (ADAMS) and other cognitive ageing studies around the world. For new collaborative projects and enquiries about data sharing, please contact Dorina Cadar at [d.cadar@ucl.ac.uk].

## Supplementary data

Supplementary data are available at *IJE* online.

## Funding

The English Longitudinal Study of Ageing Harmonised Cognitive Assessment Protocol (ELSA-HCAP) is funded by the National Institute on Aging (grant R01AG017644) and performed at the Institute of Epidemiology and Health Care, University College London. The English Longitudinal Study of Ageing is funded by the National Institute on Aging (grant R01AG017644) and by a consortium of UK government departments coordinated by the Economic and Social Research Council (ESRC) and, since 2018, by the National Institute for Health Research. The English Longitudinal Study of Ageing (ELSA) was developed by a team of researchers based at University College London, the Institute for Fiscal Studies, University of Manchester, and NatCen Social Research. The ELSA-HCAP Sub-study has been being carried out by NatCen and the Institute of Epidemiology and Health Care at University College London, with collaborators from University of Cambridge, University of Exeter and Newcastle University. The National Institute on Aging had no role in preparing this manuscript.

## Supplementary Material

dyaa227_Supplementary_Data
