# Supplementary material for: Cohort Profile Update: The Harmonised Cognitive Assessment Protocol Sub-study of the English Longitudinal Study of Ageing (ELSA-HCAP)
Source: Int J Epidemiol. 2020 Dec 28;50(3):725–726i. doi: 10.1093/ije/dyaa227 (PMC8271185; doi:10.1093/ije/dyaa227)
Supplement: dyaa227_Supplementary_Data [file dyaa227_supplementary_data.docx]

**Supplementary material**

Supplementary information on ELSA-HCAP Consent and capability

Supplementary information on ELSA-HCAP Dress rehearsal sample specification

Supplementary information on ELSA-HCAP Sample weights

Supplementary tables and figures

Supplementary Table S1: Population estimates used in calibration weighting

Supplementary Table S2. The full battery of neuropsychological tests included in the ELSA-HCAP respondent and their references

Supplementary Figure S1. The timeline of the ELSA-HCAP sub-study within the main ELSA waves

**Supplementary Material**

*Supplementary information on ELSA-HCAP Consent and capability*

Participation in the ELSA HCAP study was entirely voluntary. The purpose of the study and voluntary nature of the interview were explained in the advance letter and leaflet sent to respondent’s residence, and again at the start of the interview. The participant gave informed consent to the interviewer that they were willing to participate. The interviewers paid particular attention to the respondent’s capacity to understand and provide fully informed consent. Where interviewers suspected that a respondent was not fully capable of giving informed consent, because they appeared confused or struggled to understand what they were being asked, the interviewer contacted a family member, friend or a carer. They were asked to consider whether the respondent would be likely to agree to take part in the study if they were capable of making the decision themselves. If they felt that the respondent would be willing to participate (for example, because they had previously had conversations with them about their participation in ELSA), and they agreed that the respondent would not be harmed or distressed by participating, then the consultee signed a declaration form to give consent on the respondent’s behalf.

*Supplementary information on ELSA-HCAP Dress rehearsal sample*

The dress rehearsal sample was selected from the ELSA pilot and dress rehearsal panel, who are long-standing participants who understand that their role is to test the design, content and materials of a proposed ELSA interview. From among this group, a purposive sample was drawn of participants who had participated in the ELSA Wave 8 dress rehearsal (or Wave 7, if temporarily unavailable at Wave 8) and whose home address was within reasonable travel distance of one of the interviewers recruited. For the dress rehearsal fieldwork, 61 cases were recruited, resulting in 49 completed respondent interviews. Of those, 48 were able to nominate someone to act as their informant, and 44 of those resulted in completed informant interviews. From these, 27 of these were completed as paper self-completion questionnaires and 17 as telephone interviews.

*Supplementary information on ELSA-HCAP Sample weights*

*Design weights* were calculated by using the inverse of the probability of selection (equal to either 1 or 5) multiplied by the cross-sectional weight from the last ELSA wave in which the individual took part (e.g., wave 8 for most participants and wave 7 otherwise). The resulting weight was adjusted (at the 99th percentile) to eliminate any extreme weights.

*Non-response weights* were then modelled using logistic regression with a suite of socio-demographic and health measures (from ELSA) used as explanatory variables of the individual response to HCAP, in models weighted by the design weights. The response rates and pattern of non-response were found significantly different (*p≤0.05*) between the three sampling cognition groups (described in the eligibility criteria section above). The lowest level of response was found as expected in the low cognition group. Therefore, two sets of variables were created to allow for adjustments to be made both across and within the three cognition groups. Each variable with four or more categories was then recoded to have only two or three categories; thus, there were two versions of most explanatory variables. For example, the highest education in five categories was recoded into three categories. The original set of variables was used for main effects (e.g., adjustments for the whole sample) and the reduced set of variables for interactions (e.g., adjustments within the group). The final model included the following variables: age and cognition group, region, ethnicity, tenure, current work status, self-rated long-standing limiting illness, health status, memory, hearing, taking part in mildly energetic activities, moderately energetic activities, or strenuous activities, difficulties with daily living activities (ADL), objective measures (quartiles) of memory, verbal fluency and interactions between cognition groups with each of the age groups, tenure, self-rated memory, ADL, objective memory. A non-response weight was created by taking the inverse of the response probability from the model, trimmed at the 99th percentile, and multiplied by the selection weight.

*Calibration weighting* was then created to adjust the weights in order to match population estimates. The choice of variables used in calibration was informed by a comparison between the achieved sample (weighted by the composite non-response weight) and ELSA core members aged 65+ at wave 8 (weighted by the cross-sectional weight). Age group and sex were included in the calibration within cognition group, while region, ethnicity, tenure, education (highest qualification), marital status, self-reported health status, whether have a long-standing limiting illness, were included in the overall sample. The weights were calibrated by age and sex within each cognition group, and by education, ethnicity, and marital status across groups, although the calibration adjustment was minimal in ELSA-HCAP, meaning that the distributions of other variables used in the non-response weighting were very close to population estimates.

Population estimates (shown in Table S1) are taken from ELSA wave 8 core members aged 65+ weighted by the cross-sectional weight. The final weight for this study is a combination of the design and non-response weights. The calibration weights thereby make the weighted HCAP sample representative of the population aged 65+ in England. The unweighted results of this sample will not be the same as the population.

**Supplementary Table S1. Population estimates used in calibration weighting**

|  | **Low cognition** | **Moderate cognition** | **Normal/ unknown cognition** | **All** |
| --- | --- | --- | --- | --- |
| **Age group** |  |  |  |  |
| 65-69 | 0.5% | 2.3% | 28.7% | 31.6% |
| 70-74 | 1.2% | 3.2% | 20.4% | 24.7% |
| 75-79 | 1.4% | 2.9% | 14.2% | 18.4% |
| 80+ | 3.7% | 6.7% | 14.8% | 25.2% |
| Total | 6.9% | 15.1% | 78.1% | 100.0% |
|  | **Low cognition** | **Moderate cognition** | **Normal/ unknown cognition** | **All** |
| **Sex** |  |  |  |  |
| Male | 3.1% | 6.8% | 36.2% | 46.0% |
| Female | 3.8% | 8.3% | 41.9% | 54.0% |
| Total | 6.9% | 15.1% | 78.1% | 100.0% |
| **Region** | **All** | | | |
| North East | 5.1% | | | |
| North West | 13.3% | | | |
| Yorkshire and The Humber | 10.0% | | | |
| East Midlands | 9.1% | | | |
| West Midlands | 10.8% | | | |
| East of England | 12.0% | | | |
| London | 10.4% | | | |
| South East | 17.3% | | | |
| South West | 12.0% | | | |
| Total | 100.0% | | | |
| **Highest qualification** |  | | | |
| Degree or equivalent | 15.8% | | | |
| A level/higher education below degree | 22.9% | | | |
| O level or other | 18.5% | | | |
| CSE or other | 13.3% | | | |
| No qualifications | 29.5% | | | |
| Total | 100.0% | | | |
| **Tenure** |  | | | |
| Own/mortgage | 83.3% | | | |
| Renting | 16.7% | | | |
| Total | 100.0% | | | |
| **Ethnicity** |  | | | |
| White | 97.0% | | | |
| Non-white | 3.0% | | | |
| Total | 100.0% | | | |
| **Marital status** |  | | | |
| Single | 4.4% | | | |
| Married/civil partnership | 53.6% | | | |
| Remarried, second or later marriage | 9.6% | | | |
| Separated/divorced | 11.4% | | | |
| Widowed | 21.0% | | | |
| Total | 100.0% | | | |
| **Self-reported health status** |  | | | |
| Excellent/very good | 33.2% | | | |
| Good | 36.5% | | | |
| Fair | 21.0% | | | |
| Poor | 9.3% | | | |
| Total | 100.0% | | | |
| **Whether has a self-reported long-standing illness** |  | | | |
| Yes | 62.6% | | | |
| No | 37.4% | | | |
| Total | 100.0% | | | |

**Supplementary Table S2. The battery of tests included in the ELSA-HCAP respondent** **and informant interviews**

| **ELSA-HCAP respondent interview** |
| --- |
| Mini-Mental State Examination (MMSE)^1^ |
| HRS Telephone Interview for Cognitive Status (HRS-TICS) |
| CERAD Word List Recall–Immediate^2^ |
| Retrieval Fluency |
| Letter Cancellation |
| Backward Counting^3^ |
| 10/66 (Community Screening Instrument for Dementia, CSI-D)^4^ |
| CERAD Word List Recall–Delayed^5^ |
| East Boston Memory Test–Immediate^6^ |
| Wechsler Memory Scale-IV*–Immediate^7^ |
| CERAD Word List Recognition^2^ |
| Constructional Praxis–Immediate |
| Symbol-Digit Modalities Test^8^ |
| Constructional Praxis–Delayed |
| Wechsler Memory Scale-IV–Delayed |
| East Boston Memory Test–Delayed |
| Wechsler Memory Scale-IV–Recognition |
| Number Series^9^ |
| Raven’s Standard Progressive Matrices^10^ |
| Trail Making A & B^11^ |
| Center for Epidemiological Studies Depression Scale (CES-D)^12^ |
| Smell Test^13^ |
| **ELSA-HCAP informant interview** |
| Jorm Informant Questionnaire on Cognitive Decline in the Elderly (IQCODE)^14^ |
| Blessed Dementia Rating Scale-Part 2^15^ |
| HRS Activities Questionnaire |
| Community Screening Instrument for Dementia (CSI-D) Cognitive Activities Questionnaire^16^ |
| 10/66 Dementia Research Group Informant Questionnaire^17^ |
| Blessed Dementia Rating Scale–Part 1 |

* Wechsler Memory Scale-IV story administered in this study was ‘Anna Thompson’

ELSA-HCAP: English Longitudinal Study of Ageing-Harmonised Cognitive Assessment Protocol;

HRS: Health Retirement Study; CERAD: Consortium to Establish a Registry for Alzheimer’s Disease;


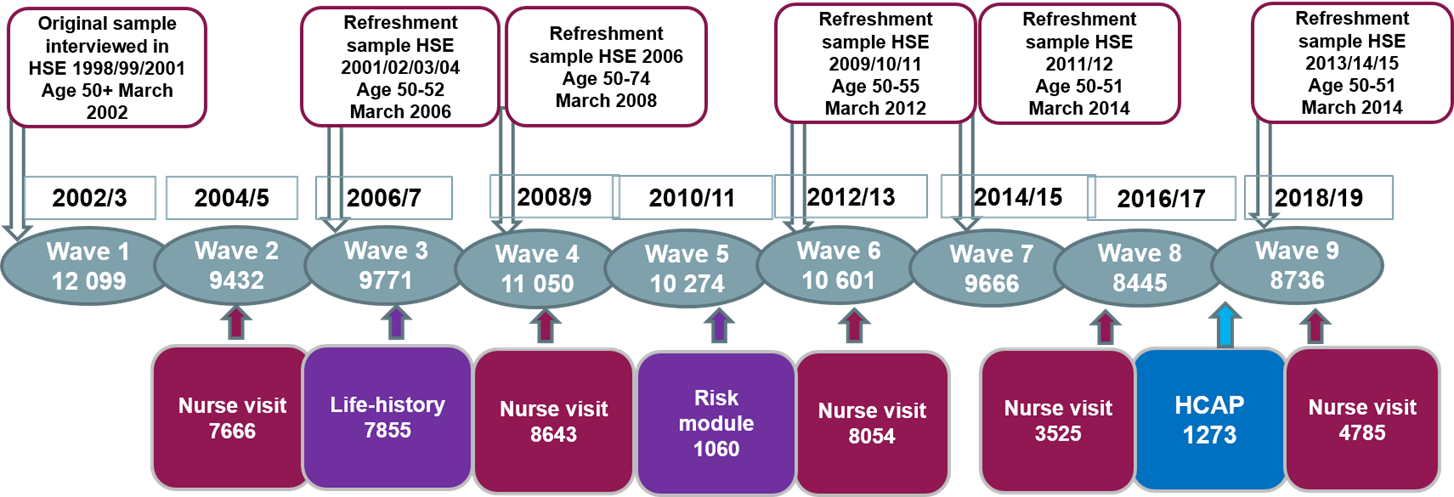


**Supplementary Figure S1. The timeline of the ELSA-HCAP sub-study within the main ELSA waves**

ELSA-HCAP: English Longitudinal Study of Ageing-Harmonised Cognitive Assessment Protocol

**References**

1. Folstein MF, Folstein SE, McHugh PR. "Mini-mental state". A practical method for grading the cognitive state of patients for the clinician. *J Psychiatr Res* 1975; **12**: 189-98.

2. Fillenbaum GG, van Belle G, Morris JC, et al. Consortium to Establish a Registry for Alzheimer's Disease (CERAD): the first twenty years. *Alzheimers Dement* 2008; **4**: 96-109.

3. Lachman ME, Agrigoroaei S, Tun PA, Weaver SL. Monitoring cognitive functioning: psychometric properties of the brief test of adult cognition by telephone. *Assessment* 2014; **21**: 404-17.

4. Prince M, Acosta D, Ferri CP, et al. A brief dementia screener suitable for use by non-specialists in resource poor settings--the cross-cultural derivation and validation of the brief Community Screening Instrument for Dementia. *Int J Geriatr Psychiatry* 2011; **26**: 899-907.

5. Fillenbaum GG, Burchett BM, Unverzagt FW, Rexroth DF, Welsh-Bohmer K. Norms for CERAD constructional praxis recall. *Clin Neuropsychol* 2011; **25**: 1345-58.

6. Scherr PA, Albert MS, Funkenstein HH, et al. correlates of cognitive function in an elderly community population. *American Journal of Epidemiology* 1988; **128**: 1084-101.

7. Wechsler D. *Wechsler Memory Scale - Revised Manual*. San Antonio: The Psychological Corporation; 1987.

8. Smith A. *Symbol digit modalities test: Manual.* Los Angeles, CA: Western Psychological Services; 1982.

9. Fisher GG, McArdle JJ, McCammon R, Sonnega A, DR W. *New Measures of Fluid Intelligence in the HRS*. Ann Arbor, Michigan: Institute for Social Research, University of Michigan; 2013.

10. Raven J. *Manual for Raven’s Progressive Matrices and Vocabulary Scales. Research supplement no. 1: The 1979 British standardisation of the standard progressive matrices and mill hill vocabulary scales, together with comparative data from earlier studies in the UK, US, Canada, Germany and Ireland.* San Antonio, TX: Oxford: Oxford University PressThe Psychological Corporation.; 1981.

11. Reitan RM, Wolfson D. Category Test and Trail Making Test as measures of frontal lobe functions. *Clinical Neuropsychologist* 1995; **9**: 50-6.

12. Radloff LS. The CES-D Scale: A Self-Report Depression Scale for Research in the General Population. *Applied Psychological Measurement* 1977; **1**: 385-401.

13. Kern DW, Wroblewski KE, Schumm LP, Pinto JM, McClintock MK. Field Survey Measures of Olfaction: The Olfactory Function Field Exam (OFFE). *Field methods* 2014; **26**: 421-34.

14. Jorm AF. A short form of the Informant Questionnaire on Cognitive Decline in the Elderly (IQCODE): development and cross-validation. *Psychol Med* 1994; **24**: 145-53.

15. Blessed G, Tomlinson BE, Roth M. The Association Between Quantitative Measures of Dementia and of Senile Change in the Cerebral Grey Matter of Elderly Subjects. *British Journal of Psychiatry* 1968; **114**: 797-811.

16. Hall KS, Hendrie HH, Brittain HM, et al. The development of a dementia screening interview in two distinct languages. *International Journal of Methods in Psychiatric Research* 1993; **3**: 1–28.

17. Prince M, Ferri CP, Acosta D, et al. The protocols for the 10/66 dementia research group population-based research programme. *BMC Public Health* 2007; **7**: 165.
